# Human papillomavirus and gastrointestinal cancer in Iranian population: A systematic review and meta-analysis 

**DOI:** 10.22088/cjim.8.2.67

**Published:** 2017

**Authors:** Versa Omrani-Navai, Reza Alizadeh-Navaei, Yousef Yahyapour, Akbar Hedayatizadeh-Omran, Saeid Abediankenari, Ghasem Janbabaei, Fatima Toghani

**Affiliations:** 1 Immunogenetic Research Center, Mazandaran University of Medical Sciences, Sari, Iran.; 2 Gastrointestinal Cancer Research Center, Mazandaran University of Medical Sciences, Sari, Iran.; 3 Infectious Diseases and Tropical Medicine Research Center, Health Research Institute, Babol University of Medical Sciences, Babol, Iran.

**Keywords:** Gastrointestinal cancers, Human papillomavirus, Systematic review, Meta-analysis

## Abstract

**Background:**

Gastrointestinal (GI) malignancies are the most common cancers and account for nearly half of all cancer-related deaths in Iran. There was a strong association between human papillomavirus (HPV) infection and urogenital cancers, in particular the cervix. However, there is no clear causal relationship in all types of cancers, including gastrointestinal cancers. Therefore, the present study as a systematic review and meta-analysis was designed to evaluate the prevalence and relation of HPV in GI cancers.

**Methods:**

This systematic review and meta-analysis study assess the prevalence of human papillomavirus in GI cancers in Iran. Data were collected by searching electronic databases, including PubMed, Google Scholar, Scopus, SID and Iranmedex by English and Persian key words up to August 2016. Key words included: Human Papillomavirus, HPV, Cancer, Neoplasm, Carcinoma, Esophageal, colorectal, Gastrointestinal and Iran articles were entered in the EndNote software and duplicate papers were excluded. Data were extracted and analyzed by comprehensive meta-analysis software, Version 2 (CMA.V2) and random effects model.

**Results:**

Finally, we included 17 studies in this meta-analysis. The prevalence of HPV in Iranian patients with GI cancers was 16.4% (CI95%: 10.4-24.9). Considering all HPV types, the odds ratio of GI cancers in positive patients was 3.03 (CI95%: 1.42-6.45) while in patients with HPV-16 was 3.62 (CI: 1.43-4.82).

**Conclusion:**

The results show a strong relationship between HPV infection especially high-risk HPV type 16 and GI cancers in Iranian population.

Asian countries such as Iran are encountering increased prevalence and mortality caused by cancer and ranks third as the cause of death after cardiovascular disorders and road traffic injuries (1). Due to demographic changes and increasing life expectancy in Iran, it has been estimated that the incidence of cancer increases from 84,800 cases in 2012 and will rise to 129,700 in 2025 (a 35% increase) (2). This rapid development of different types of cancer has directed the medical scientists and researchers to answer a variety of questions about the sources and reasons of this disease. The increased middle-aged and elderly population, advancements in the new diagnostic tools and screening as a standalone test, cannot be a proper justification for the rising growth of this disease in different societies around the world (3, 4). This issue has remarkably highlighted the other causing factors as the new sources of this disease as lifestyle-related, genetic factors and disease-promoting factors, like microorganisms (bacteria, parasites particularly viruses) (5). It is estimated that 15 to 20% of all cancers may be attributed to viral agents (6-9). 

HPV as well as H. pylori, hepatitis B and C viruses are among the well-known that causes cancer infectious agents (10, 11). Primary infection of HPV is acquired through contact with skin lesions, which is usually detected and eliminated by the immune system. In case of persistent infection, virus integrates into host DNA and through increase cell proliferation and disruption, tumor suppressor pathways facilitate the development of cancer (12). HPV develops a range of epithelial hyperplasia and depending on the strength of lesion in the creation of progressive malignancy, two mucosal and cutaneous groups are classified. Mucosal low-risk HPV such as HPV6 and HPV11 create genital warts, while high-risk types like HPV16 and HPV 18 can lead to squamous intraepithelial lesions which can progress to invasive squamous cell carcinoma stage (13). 

In 2013, it was stated that of the 12.7 million cancers in the world, 4.8% (about 610,000 cases) may be attributed to HPV. This statistics ranges from 15.5% in India to 1.2% in Australia (14). 

HPV is a well-established risk factor for developing urogenital/ oropharynx cancers in particular the cervix (76% in Iranian patients) (15). Nonetheless, there is no clear causal relationship in all types of cancers, including cancers of the digestive (gastrointestinal) system. GI malignancies are the most common cancers and account for almost half of all cancer-related mortality in Iranian population. In the meantime, gastric, esophageal and colorectal cancers are parts of the five most prevalent cancers in Iran (1, 16). Multiple risk factors are involved in the development of these malignancies such as family history, smoking, consumption of alcohol and hot foods, genetic mutations and infections. HPV oncogenic types have been detected in esophageal and colorectal cancers but studies on this topic are conflicting (1). 

The present range of HPV DNA in colorectal and esophageal cancers in Iran is reported between 0 -34.2% (17, 18) and 0-37.5% (19, 20), respectively. Thus, according to the conflicts and the absence of a comprehensive review about the prevalence and association between HPV infections and GI cancers, we aimed to perform a systematic review and meta-analysis on this issue.

## Methods

**Literature search strategy: **This systematic review and meta-analysis investigated the prevalence and relation between HPV infection and GI cancers in Iran. International electronic databases; PubMed, Scopus, Google Scholar and National databases including Scientific Information Database, Iranmedex, and Magiran were systematically searched up to August 2016. Key words were selected according to medical subheading (MeSH) with all possible meaningful combinations including; Colorectal, Neoplasm, Esophageal, Cancer, Human papilloma virus affiliated to Iran and appropriate equivalents in Farsi. 

**Inclusion and exclusion criteria: **All case-control and cross-sectional studies conducted on HPV infection in GI cancers affiliated to Iran were included in the research. Other article types including case report/series, letter to editor and review were eliminated. Studies that examined HPV seropositivity or detection techniques other than polymerase chain reaction (PCR) were eliminated as well.

**Study Selection: **Selected articles were assessed by two researchers independently. Initial abstracts and full texts were evaluated. A possible discrepancy between the reviewers was resolved by discussions.

**Study Quality Control: **Diversified aspects of the methodology including type of survey, measurement and diagnostic methods and statistical analysis were evaluated according to the Strobe checklist.

**Data Extraction: **According to the prepared checklist, author name, year of study, type of cancer, type of specimen, number of patients, number of healthy controls (in case-control studies), number of positive samples in each group and detected HPV type were extracted.

**Data Analysis: **Finally, data were analyzed using the comprehensive meta-analysis software version 2.0 and random effects method. Forest diagrams were plotted for the prevalence of HPV in GI cancer and the relationship between HPV and GI cancers. Publication bias examples were evaluated by Funnel plot and were drawn for the relationship between HPV and GI cancers. Heterogeneity was checked by I2 index. 

## Results

**Figure 1:**
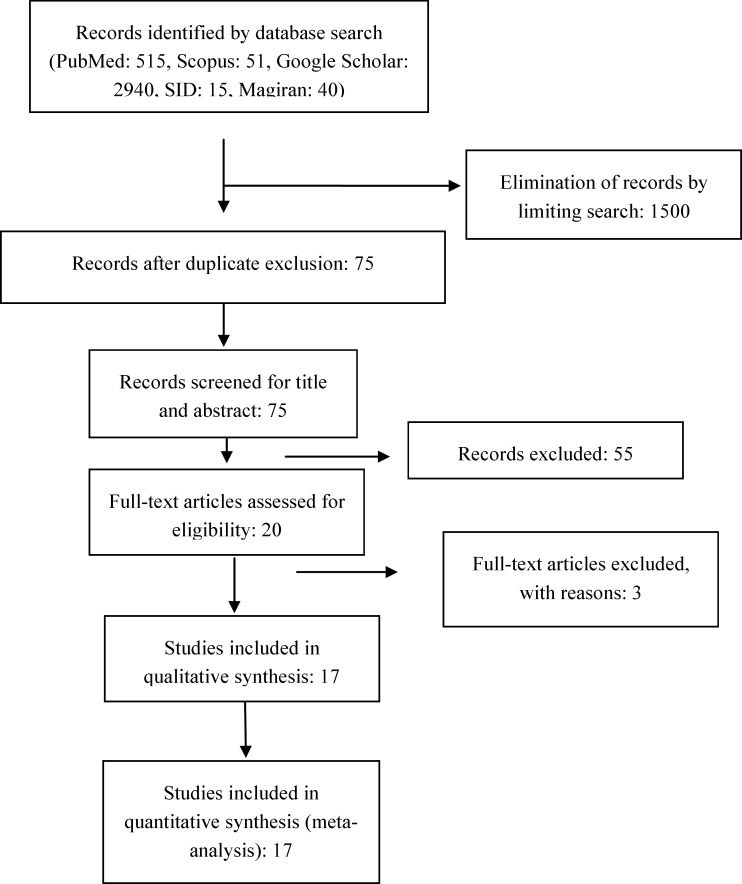
PRISMA diagram of study identification and selection

With all possible keyword combinations, our primary search in mentioned databases yielded 3561 articles (figure 1). After removing and eliminating duplicate records by limiting search, two authors (R.A and V.O) independently evaluated the abstracts and full texts of the remaining 75 articles. In this part, papers using diagnostic methods other than PCR (i.e. serology-based), review, case report/series and studies lacking the required qualities were excluded. Finally, we entered 17 studies in this meta-analysis conducted during 2005-2016.

**Figure 2:**
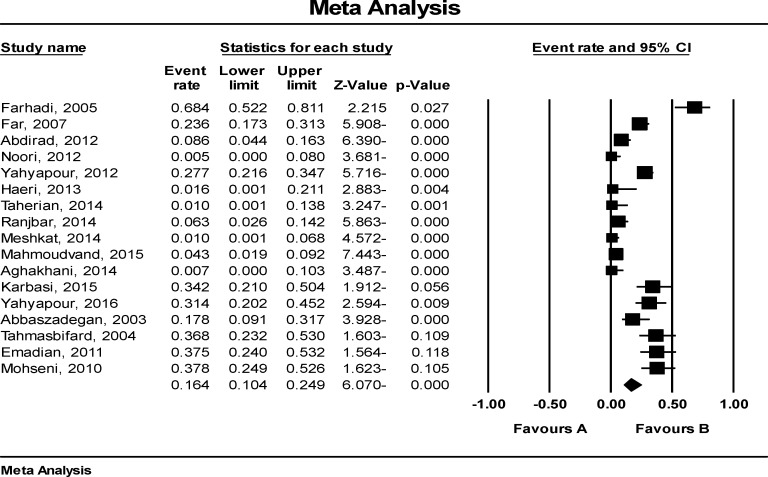
Forest plot of HPV prevalence in Iranian patients with GI cancers

A total of 1337 patients and 561 controls were investigated to assess the prevalence and the relationship between HPV and GI cancer. Features of study participants are shown in table 1. Studies were carried out in five different provinces in Iran: Tehran (21), Mazandaran (12), Mashhad (20), Shiraz (2) and Gilan (1). All of the specimens were paraffin-embedded tissues with the exception of one study (fresh frozen). As shown in figure 2, the prevalence of HPV in Iranian patients with GI cancer was 16.4% (CI 95%: 10.4-24.9). 

**Figure 3:**
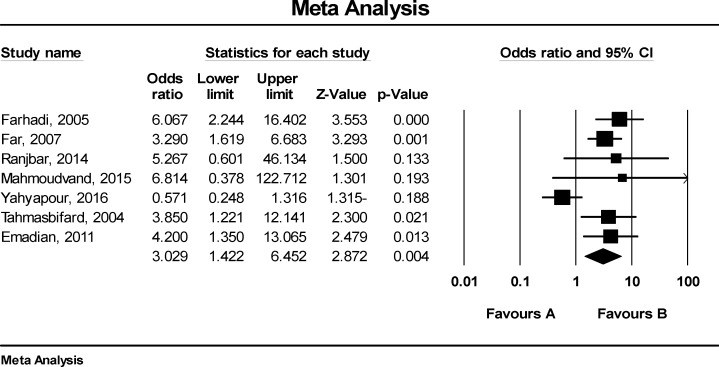
The relationship between HPV and GI cancers in Iranian patients

**Figure 4:**
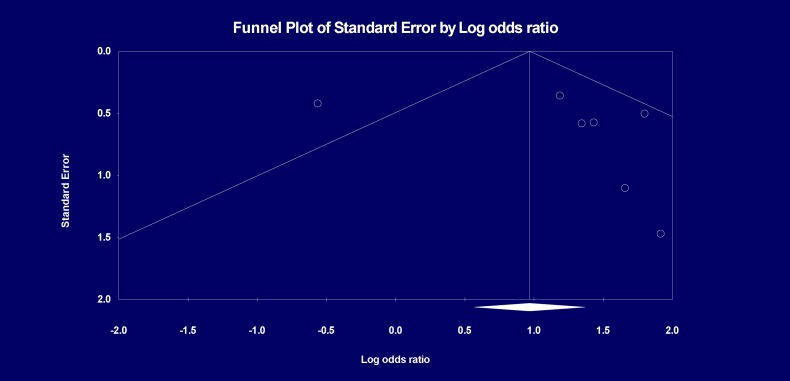
Funnel plot of the relationship between HPV and GI cancers in Iranian patients

**Figure 5:**
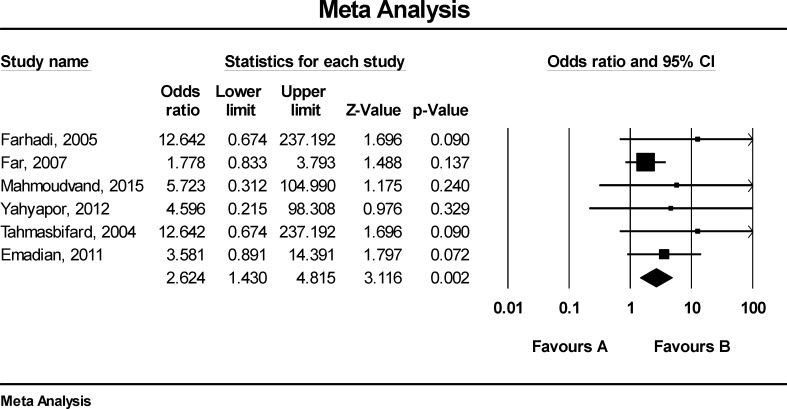
The relationship between HPV-16 and GI cancers in Iranian patients

Figure 3 shows that the odds ratio (OR) for GI cancers in HPV positive patient was 3.03 (CI95%: 1.42-6.45). According to figure 4, there was publication bias in the reviewed studies between HPV with GI cancer in Iranian patients. Figure 5 shows that the OR for GI cancers in patients with HPV16 was 3.62 (CI: 1.43-4.82). 

**Table 1:** Characteristics of studies enrolled in the systematic review and meta-analysis investigating the prevalence and association between HPV infection and GI cancers

**Author**	**Province**	**Year of publication**	**Cancer type**	**Specimen**	**No. case**	**No. positive**	**No. ctrl**	**No. positive**	**Detection method**	**HPV-Type detected**
Abbaszadegan et al. (20)	Mash’had	2003	esophageal squamous cell carcinoma (ESCC)	paraffin blocks	45	8	-	-	HPV16/18: E6/E7	HPV16
Tahmasebi Fard et al.(21)	Tehran	2004	esophageal squamous cell carcinoma (ESCC)	paraffin blocks	38	14	38	5	L1 gene: MY09/MY11 HPV16/18: E6/E7	HPV16,18
Farhadi et al. (22)	Tehran	2005	esophageal squamous cell carcinoma (ESCC)	paraffin blocks	38	14	38	5	L1 gene: MY09/MY11 HPV16/18: E6/E7	HPV18,16
Far et al. (23)	Tehran	2007	esophageal squamous cell carcinoma (ESCC)	paraffin blocks	140	33	140	12	L1 gene: GP5+/GP6+ sequencing	HPV16,18,33 and 31
Mohseni et al. (24)	Gilan	2010	esophageal squamous cell carcinoma (ESCC)	paraffin blocks	45	17	-	-	L1 gene: GP5+/GP6+ genotyping	HPV16,18, 31,33,51,52,58
Emadian et al. (19)	Mazandaran	2011	esophageal squamous cell carcinoma (ESCC)	paraffin blocks	40	15	40	5	PCR, genotyping by Kit	HPV16,45
Abdirad et al. (25)	Tehran	2012	esophageal squamous cell carcinoma (ESCC)	paraffin blocks	93	8	-	-	L1 gene: SPF10 sequencing	HPV6,16 and 18
Noori et al. (18)	Shiraz	2012	esophageal squamous cell carcinoma (ESCC)	paraffin blocks	92	0	-	-	L1 gene: GP5+/GP6+	-
Yahyapour et al. (26)	Mazandaran	2012	esophageal squamous cell carcinoma (ESCC)	paraffin blocks	177	49	-	-	L1 gene: MY09/MY11	-
Haeri et al. (27)	Tehran	2013	esophageal squamous cell carcinoma (ESCC)	paraffin blocks	30	0	30	0	L1 gene: GP5+/GP6+	-
Yahyapour et al. (28)	Mazandaran	2016	esophageal squamous cell carcinoma (ESCC)	paraffin blocks	51	16	45	20	L1 gene: MY09/MY11 genotyping	HPV11,16, 33,35,39,45, 52,56 58 and 59
Taherian et al. (29)	Tehran	2014	Colorectal cancer	paraffin blocks	50	0	50	0	L1 gene: MY09/MY11	-
Ranjbar et al. (30)	Tehran	2014	colorectal cancer	paraffin blocks	80	5	80	1	L1 gene: MY09/MY11, GP5+/GP6+ sequencing	HPV18
Meshkat et al. (31)	Mashhad	2014	colorectal cancer	paraffin blocks	100	1	-	-	L1 gene: GP5+/GP6+	
Aghakhani et al. (16)	Tehran	2014	Colon adenocarcinoma colon adenoma		70 70	0 0	30	0	L1 gene: MY09/MY11, GP5+/GP6+	-
Mahmoudvand et al. (32)	Shiraz	2015	colorectal adenocarcinoma colorectal adenomatous polyps	paraffin blocks	70 70	2 4	70	0	L1 gene: MY09/MY11 HPV16/18: E6	HPV16,18
Karbasi et al. (17)	Tehran	2015	colorectal cancer	fresh frozen tissue	38	13	-	-		HPV16,18

## Discussion

The aim of this systematic review and meta-analysis was to determine the prevalence of HPV in gastrointestinal cancers and its relation as a risk factor for these cancers. Result of the present study shows that the prevalence of HPV in Iranian patients with GI cancer was 16.4% and it seems that this rate is higher than the expected value in Bucchi’s review study which reported that the exposure to HPV is very common and the majority of HPV infections are asymptomatic. Nevertheless, there is a 10% chance that individuals can develop a persistent infection and may have an increased risk of developing a cancer (33). 

In the present study, the OR of getting cancer in Iranian patients with HPV positive was 3 times more than HPV negative. This rate for HPV16 was 3.6, in other words, there was a strong association between gastrointestinal cancer and infections with HPV particularly HPV16 in Iranian population. A meta-analysis to assess HPV-16/18 in esophageal cancer was performed by Yong et al. Of the 5755 cases, 11.67% and 1.82% harbored HPV-16 and 18, respectively. The OR reported for HPV-16 and risk of cancer (3.55) is quite similar to ours (34). Zhang et al. investigated the association between HPV-16 and esophageal cancer in Chinese population and reported that being infected with HPV-16 increases the chance of esophageal squamous cell carcinoma more than six folds (35). About one-third of samples they analyzed were fresh frozen biopsies which increase the chance of HPV detection. Also, higher OR can be attributed to differences in target populations (36). Li et al. in 2014 carried out a similar meta-analysis on HPV infection and esophageal squamous cell carcinoma and adenocarcinoma worldwide. Overall, the prevalence of HPV was 22.2% in SCC and 35% in adenocarcinoma. In line with our results, the analysis association for HPV-16 and SCC showed an OR of 3.52. This incidence in Asia was approximately two times more than America and Europe (37). 

In terms of colorectal cancer in the study of 2630 cases of colon adenocarcinoma, the total prevalence of HPV was 11.2% and the obtained OR of case-control studies was about 6 (38). In contrast to our findings, the most common HPV type found by Damin et al. in Asia was HPV18. As there were fewer case-control reports positive for HPV-18, we did the type specific analysis for HPV-16 only. In addition, the overall incidence and OR in mentioned studies was considerably higher than our results (31.9% and 10.04% respectively). This conflicting result can be affected by the higher incidence of infections in South America (34). Although it has been more than 30 years since the introduction of HPV infection in GI cancers, no strong evidence has been proven with regard to the role of this virus in the mentioned cancers. The pattern of HPV infection is globally diverse and ranges from more than 80% in China to 0% in Brazil (39). The studies enrolled in the present meta-analysis had contrary reports, too. Noori and Haeri with regard to esophageal cancer reported no HPV infection, similar to Taherian and Aghalshai regarding colorectal. High HPV-positive sample was seen in Mohseni et al;s study in the North of Iran considered as a high risk area for gastric cancer (table 1). The differences in diagnostic methods may be responsible for diverse reports of HPV infection; consequently, we included published studies with a similar technique. Accordingly, it is possible that geographic differences in HPV distribution patterns and the existence of other risk factors contributing to HPV-associated cancers be involved in this issue. Hence, it is necessary that future studies in Iran consider the risk factors for GI cancers more precisely. 

Another point that should be kept in mind is the existence of HPV DNA which does not indicate an active infection. Most HPV infections are spontaneously eliminated by immune response within one year, and only a small proportion of them established transformed infections. As a result, research targeting viral oncogenes (E6 and E7) through ribonucleic acid (RNA) and protein level can provide more accurate information (40, 41). A (other) meta-analysis done investigating HPV in breast and cervical cancers in Iran has similar outcomes (42, 43) It appears that HPV increased the risk of certain cancers in Iranian population. 

As limitations of the current study, all of the ORs extracted from primary study had crude rate and there were no adjusted rates in all studies. Moreover, the strength of the present study as far as we know, is its being the first systematic review and meta-analysis that aimed to investigate the prevalence and relation between HPV and GI cancers in Iranian population. In conclusion, the results of our study indicate that there is a strong relationship between gastrointestinal cancer and HPV infection particularly the high risk HPV type 16 in Iran. HPV screening and preventive vaccine-based policies in high-risk groups need further investigations that should be considered.
